# Performance Analysis of Retrofitted Tribo-Corrosion Test Rig for Monitoring In Situ Oil Conditions

**DOI:** 10.3390/ma10101145

**Published:** 2017-09-28

**Authors:** Arpith Siddaiah, Zulfiqar Ahmad Khan, Rahul Ramachandran, Pradeep L. Menezes

**Affiliations:** 1 Mechanical Engineering Department, University of Nevada, Reno, NV 89557, USA; asiddaiah@nevada.unr.edu (A.S.); rramachandran@unr.edu (R.R.); 2 Department of Design & Engineering, NanoCorr, Energy & Modelling (NCEM), Bournemouth University, Fern Barrow, Dorset BH12 5BB, UK; zkhan@bournemouth.ac.uk; 3 Nevada Institute for Sustainability, University of Nevada, Reno, NV 89557, USA

**Keywords:** tribo-corrosion, lubricants, in situ lubricant monitoring, pin-on-disk retrofit, friction, corrosion, wear

## Abstract

Oils and lubricants, once extracted after use from a mechanical system, can hardly be reused, and should be refurbished or replaced in most applications. New methods of in situ oil and lubricant efficiency monitoring systems have been introduced for a wide variety of mechanical systems, such as automobiles, aerospace aircrafts, ships, offshore wind turbines, and deep sea oil drilling rigs. These methods utilize electronic sensors to monitor the “byproduct effects” in a mechanical system that are not indicative of the actual remaining lifecycle and reliability of the oils. A reliable oil monitoring system should be able to monitor the wear rate and the corrosion rate of the tribo-pairs due to the inclusion of contaminants. The current study addresses this technological gap, and presents a novel design of a tribo-corrosion test rig for oils used in a dynamic system. A pin-on-disk tribometer test rig retrofitted with a three electrode-potentiostat corrosion monitoring system was used to analyze the corrosion and wear rate of a steel tribo-pair in industrial grade transmission oil. The effectiveness of the retrofitted test rig was analyzed by introducing various concentrations of contaminants in an oil medium that usually leads to a corrosive working environment. The results indicate that the retrofitted test rig can effectively monitor the in situ tribological performance of the oil in a controlled dynamic corrosive environment. It is a useful method to understand the wear–corrosion synergies for further experimental work, and to develop accurate predictive lifecycle assessment and prognostic models. The application of this system is expected to have economic benefits and help reduce the ecological oil waste footprint.

## 1. Introduction

**Figure 1:**
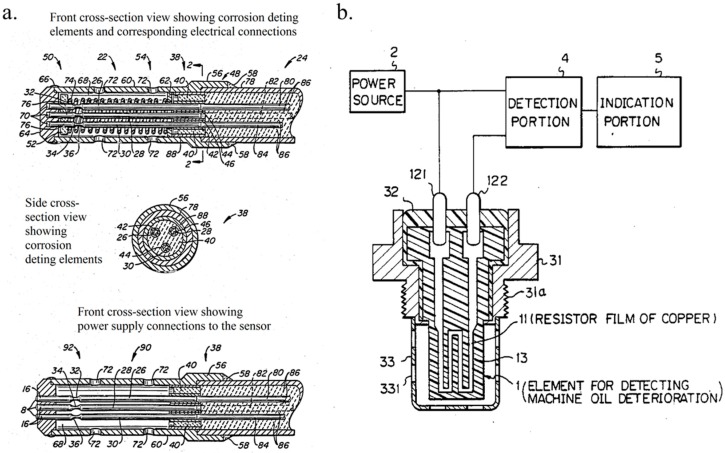
The very first fluid monitoring electrical sensors. (**a**) General Electric’s corrosion impurity sensor * [6]; (**b**) Nippon Soken Inc. machine oil deterioration detection sensor * [7]. (* Images as filed in their respective patents. The numbers indicate components of sensor specific to the filed patents).

An oil or lubricant once extracted after use can rarely be reused and should be refurbished or discarded in most cases. Mechanical systems such as automobiles, aircrafts, ships, offshore wind turbines, deep sea oil drilling rigs, and a wide variety of machinery involve the use of one or more forms of oils. While these fluids are performing their functionality of providing required boundary lubrication, their efficient functioning life has been usually estimated by a “thumb-rule” that was related to their cycles or period of usage. Unless an oil sample was removed and investigated by using several analytical techniques specific to the application [1,2], the exact remaining effective lifecycle of the fluid is difficult to be accurately estimated [3,4,5]. Over the past few decades, several ingenious methods have emerged for oil monitoring systems, which have been spearheaded by industrial research and development (R & D) [6,7,8,9]. One of the earliest forms of an electrical sensor-based fluid monitoring system is schematically represented in Figure 1a [6]. This kind of a fluid monitoring system was used for monitoring the core cooling water of a nuclear fusion light water reactor (LWR). These systems made it possible to detect at least one corrosive impurity in a fluid in situ. This was a significant technological progress when observed from the perspective of fluid quality monitoring. Further, the idea of these electrical-based sensors for fluids was later used for detecting machine oil deterioration, including the engine oil of an automobile, as schematically represented in Figure 1b [7]. This sensor involved a resistor coated with a thin copper film that corroded in the presence of acid content in the machine oil, thus indicating the extent of oil deterioration.

**Figure 2:**
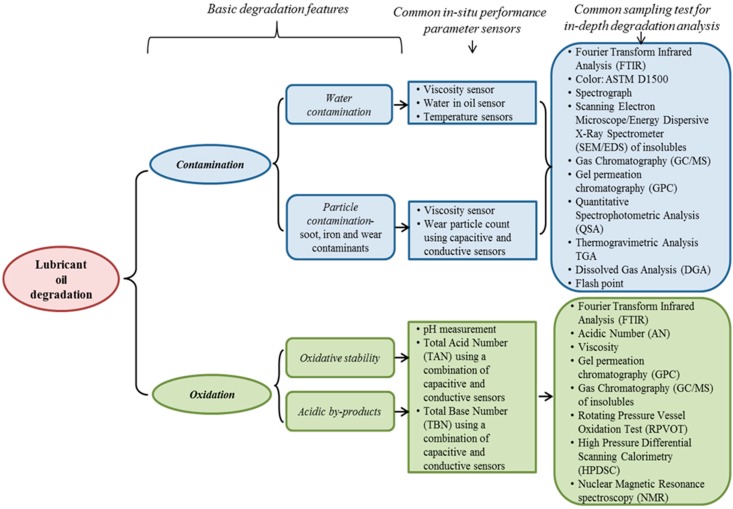
Lubricant oil degradation features and their common in situ and sampling methods of testing.

The invention of such in situ fluid monitoring sensors was capitalized in no time, and research pertaining to the integration of these sensors into mechanical systems and techniques to manufacture them on a large scale started to gain pace. This was evident from the number of high valued patents filed in 1987–1988, which tried to integrate and improve these sensors within mechanical systems found in automobile and machine components [8,9]. Though many of these corrosion-based sensors were more appropriate ways of monitoring oil condition, these systems were not easily integrated into a dynamic system. In vivo monitoring of fluid conditions in a dynamic environment using corrosion-based sensors posed a variety of abnormal problems for commercial implementation. These problems resulted in a very low current density due to the type of fluid medium, wear debris, and various contaminants accumulated in the fluid used in the mechanical system [10,11,12]. Due to the instability of integrating corrosion-based oil monitoring systems for commercial use [10], a new variety of electrical oil monitoring systems based on user handling and oil working conditions were introduced. In an engine of an automobile, these systems would simultaneously monitor the oil temperature, running time, elapsed time since the last oil change, and other such oil working conditions to mathematically predict the deterioration rate and remaining oil working lifecycle [13,14,15,16,17,18,19,20,21,22]. Some of the basic sensors and sampling test methods in relation to their lubricant oil degradation features are represented in Figure 2.

Degradation of oil is the consequence of foreign contaminants introduced in the oil medium over its functioning period. Most of these contaminants are introduced to the oil medium through wear debris generated by one or more tribo-pairs in contact within the oil medium, along with other process by-products such as water and soot [23,24,25,26]. The extent of wear occurring is defined by the type of oil, boundary lubrication, and the working environment. Further, as the contaminants enter the oil medium, they accelerate the oil degradation process linearly or sometimes exponentially with time due to the initiation of corrosion [26,27,28,29]. The degradation process of oil within an enclosed mechanical system is directly correlated to the extent of tribo-corrosion, which is the deterioration effect produced because of wear and corrosion occurring simultaneously in the oil medium. Even though the oil degradation rate can be predicted by monitoring the “by-product effects” of tribo-corrosion, such as oil temperature, cycle time, pressure, the elapsed time since the last oil change and other such oil working conditions, it is still not accurate enough to provide the actual effects of contaminants due to tribo-corrosion [9,29,30]. As can be observed, there are too many “by-product effects” of oil degradation that vary continuously. Hence, the current process of electronically monitoring all of these variables and using this data to predict the overall degradation of oil makes the estimations much more complex and unreliable in some cases [24,29,31,32,33,34,35,36,37]. The latest developments in data processing and modeling techniques include elaborate and complex corrosion predictive models that enable efficient corrosive species detection [38,39,40,41]. A wide range of corrosion species, such as pitting corrosion, crevice corrosion, stress corrosion cracking, and general forms of chemical-induced corrosion can be detected using these models. However, these models need to have an effective detection system that can provide in situ data acquisition more accurately than electrical sensor-based systems.

This is the technological gap to be addressed in oil condition monitoring systems, which are required to be able to constantly monitor the *origins* of oil degradation, i.e., wear and corrosion. If an oil monitoring system can monitor the wear rate of the tribo-pairs in contact along with the corrosion rate of the oil arising due to the contaminants, this system can be expected to be more reliable than the multitude of varieties of electronic-based oil monitoring systems. This system can be further integrated with the latest corrosion and wear analysis models to provide for condition-based predictive and prognostic outputs. The current study addresses this technological gap, which has very scarcely been addressed for an oil-lubricant medium [42,43,44,45], and presents an original design of a tribo-corrosion test rig. This test rig integrates the wear analysis of a tribo-pair in an oil medium with a three-electrode potentiodynamic corrosion cell. The test rig can monitor the corrosion rate and the wear rate of the tribo-pairs in situ. In the following section, we present a novel design of the test rig. Later, this test rig was used to perform a series of tribo-corrosion tests using industrial grade transmission oil under various contamination conditions. Finally, the results were analyzed to demonstrate the capabilities of the test rig.

## 2. Design and Experimentation

A tribometer test rig retrofitted with a Direct Current (DC) corrosion cell was used to perform in situ analysis on the effect of tribo-corrosion in an oil medium. Experiments were performed in accordance with the ASTM standards G119-04 to determine the synergistic effects of wear and corrosion.

### 2.1. Retrofitting of Ball-On-Disk Test Rig

**Figure 3:**
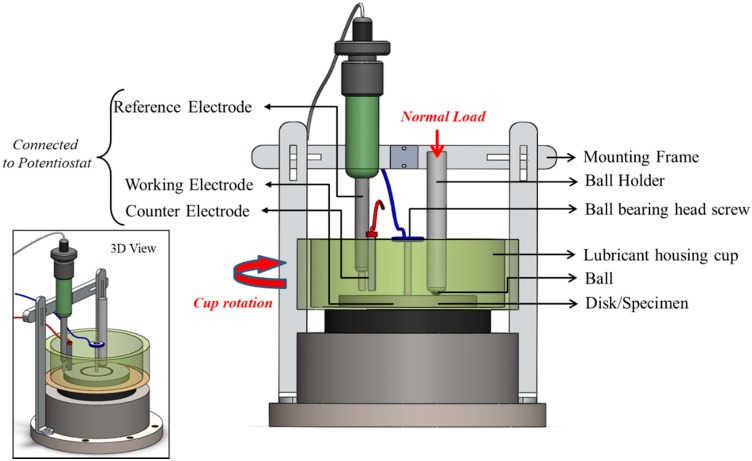
Schematic of ball-on disk test rig retrofitted with a three-electrode corrosion cell.

A multi-functional tribometer (Rtec MFT 5000, Rtec Instruments, San Jose, CA, USA) consisting of a ball-on-disk test rig with lubricant housing was retrofitted with a three-electrode corrosion cell, as represented schematically in Figure 3. The test rig consists of a rotary lubricant-housing cup containing a disk (specimen) in contact with a ball fully immersed in an oil medium. Around this lubricant housing, an adjustable mounting frame was constructed to hold the reference and the counter electrodes immersed in the oil medium, which was then connected to a potentiostat. The working electrode (which is the disk/specimen of interest) was fixed inside the lubricant housing cup using a galvanized screw with a ball-bearing bolt head extending out from the cup connecting to the potentiostat. The system contact was isolated in three ways during experimentation: the lubricating cup was made up of a non-conductive polymeric material; the experiments were conducted with a thin ceramic plate under the steel disk (working electrode); and the collects used inside the ball holder that isolated the alloy steel ball from the holder were non-metallic.

This retrofitted test rig design aims to address two major shortcomings of the tribo-corrosion set-ups currently used in lubricant oil environments. Firstly, due to the low conductivity of many oils and lubricants, the electrochemical cells for a tribo-corrosion test rig are found to be very unstable. Hence, the first challenge was to ensure accurate readings of effective resistance in the electric circuit arising from the combined effects of ohmic resistance and reactance. This is to say that the high or low impedance that may be observed in the lubricant housing cup needs to be consistently and accurately measured. This is further complicated by the fluid medium being constantly agitated in tribo-corrosion testing. To overcome this shortcoming, the counter electrode was placed as close to the reference electrode as possible, while keeping the counter electrode along the fluid flow direction for minimal flow disruption. Further, specific types and shapes of electrodes were used: a spectroscopic grade graphite counter electrode was redesigned and machined to have a droplet-shaped end so as to ensure that the maximum surface area of the counter electrode was in contact with the oil; and one of the most robust forms of reference electrode, a Saturated Calomel reference electrode (SCE) was chosen as the most suitable reference electrode for the current application.

Secondly, it is not easy to measure the potential of the system in contact accurately when both the working electrode (disk/specimen) and the conducting medium (oil) are constantly in motion. This interference was minimized by ensuring a close proximity of the reference electrode to the wear track of the ball-on-disk test. Achieving a stable setup that can provide accurate and reliable readings during the tribo-corrosion test was the first objective of this research, followed by stability testing and analysis.

### 2.2. Materials

Ball-on-disk tests were performed using 52,100 alloy steel balls of 6.5 mm diameter, and AISI 1020 steel disks of 50.8 mm diameter. The disk material used for experimentation is most commonly used as alloy structural steel in ships, vehicles, railways, bridges, and more specifically in power generation applications, mechanical gear and cam shafts that are exposed to rigorous tribological environments [46,47,48,49,50,51]. With the aim of analyzing the effectiveness of the retrofitted tribo-corrosion test rig for oil-based environments, an oil intensive system such as an automobile transmission box was selected as a reference system. The tribo-pair was tested in commercial automotive transmission oil, which meets most commercial automatic transmission fluid (ATF) specifications, as shown in Table 1.

Today, ATFs are some of the most complicated lubricating fluids that are tribologically versatile in properties and specific to applications. These ATFs must be compatible with all of the transmission components and perform consistently at both low and high temperature extremes. Hence, this system utilizes one of the most technologically advanced forms of lubricating fluids, and will provide for a distinctive idea of the tribo-corrosion test rig performance when contaminants are introduced to the lubricating medium.

### 2.3. Experimentation

Tribo-corrosion experiments under cathodic and anodic polarization conditions were performed using the retrofitted test rig, as shown in Figure 3, according to the test parameters listed in Table 2. The working electrode sample (disk) with a surface area of 2027 mm^2^ was exposed to the commercial grade lubricating medium at room temperature. Saturated calomel and graphite were used as the reference and counter electrodes, respectively. These experiments were aimed at analyzing the effectiveness of the retrofitted tribo-corrosion test rig, and investigating the tribo-corrosion in oil medium.

The experiments were performed in four stages, as listed in Table 3. Each of these experiments consisted of a specific form of boundary condition, according to ASTM G119-04 standards. Each experiment was performed for 15 h without interruption, during which the coefficient of friction (COF), wear volume, and corrosion rates were continuously monitored. The wear displacement data (change in Z-height of the ball holder) was recorded throughout these experiments, and was used to compute the specific wear rate coefficient of the system (Equation (1)). Due to the low load conditions and the wear resistant characteristics of the alloy steel ball, there was minimal contribution of the wear on the ball surface to the Z-height. The computations were based on a system of sphere-on-plane Hertzian contact pressure.

(1) $$k_{i}=\frac{h_{i}}{ps}$$

where, *h_i_* = wear displacement (using Z-height data), mm^3^; *k_i_* = specific wear rate coefficient; *p* = contact pressure = *F/A_wear track_*, N; *s* = slide distance = slide rate × time = *v* × *t*, m.

A sequence was programmed on the potentiostat to monitor the open circuit potential (OCP, *E_OC_*) throughout the tribo-corrosion experiment. Before the start of each experiment, the sample was ultrasonically conditioned by using acetone. Once dried by a blower, it was then kept in the required combination of oil medium for 1 h to obtain a stable OCP, which was followed by the 15 h tribo-corrosion experiment. An anodic condition of +0.5 V potential vs. OCP (*E_OC_*) was applied to the system in contact to accelerate the corrosion process. The cathodic condition was maintained at −0.5 V potential vs. OCP (*E_OC_*) to suppress the corrosion process. Throughout these cell conditions, the evolution of the current was monitored in parallel with the wear process. After the experimentation, the samples were left in the oil medium for 1 h so as to allow for surface repassivation. The sample was then degreased by using N-hexane and prepared for analysis of the wear track under a 3D optical profilometer and scanning electron microscope (SEM, JEOL USA Inc., Peabody, MA, USA). The tribo-corrosion experiments were performed in three conditions of oil medium listed in Table 4. All of the experiments under each oil condition were performed twice to ensure the repeatability of obtained results. The synergism of wear and corrosion has been analyzed in each of these three oil conditions.

## 3. Results and Discussion

Each stage of the experiment detailed in Table 3 was performed, and the results are discussed and analyzed in this section. Stage I of the experiment was used as the baseline to compare the wear behavior of the tribo-pair against the corrosive influence of the oil contaminants tested in Stages II–IV. All of the electrochemical experiments were performed as DC corrosion tests in the potentiodynamic state, as the current study concerns analyzing the influence of cell solution (oil medium) on the phenomenon of tribo-corrosion with no coatings on the surface of the sample involved. It is a common knowledge in the electrochemical analysis that the impedance of the resistor (oil medium) is independent of the frequency, and the current stays in phase with the voltage through this resistor. Hence, the potentiodynamic analysis was found to be appropriate for the present study, which utilizes a single frequency.

### 3.1. Open Circuit Potential (OCP)

**Figure 4:**
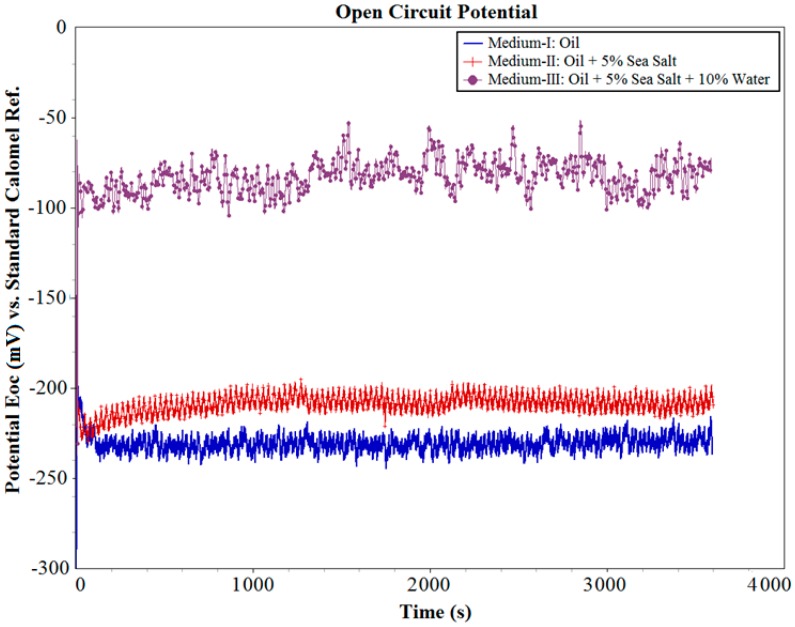
Open circuit potential (OCP) of sample in different oil mediums.

The OCP (*E_OC_*) i.e., the equilibrium (corrosion) potential of the system in contact, in each of these three oil mediums was initially measured for 1 h (3600 s), as shown in Figure 4. It can be observed that the system was able to reach a stable equilibrium potential in all three oil mediums. The OCP of the system in Medium-I was found to be −232 mV; in Medium-II was found to be −210 mV; and in Medium-III was found to be −84 mV. These values of OCP are initial indicators that even without the influence of wear, the system in Medium-II and Medium-III containing contaminants will be highly susceptible to corrosion when an electrical potential is applied.

### 3.2. Influence of Wear on OCP

**Figure 5:**
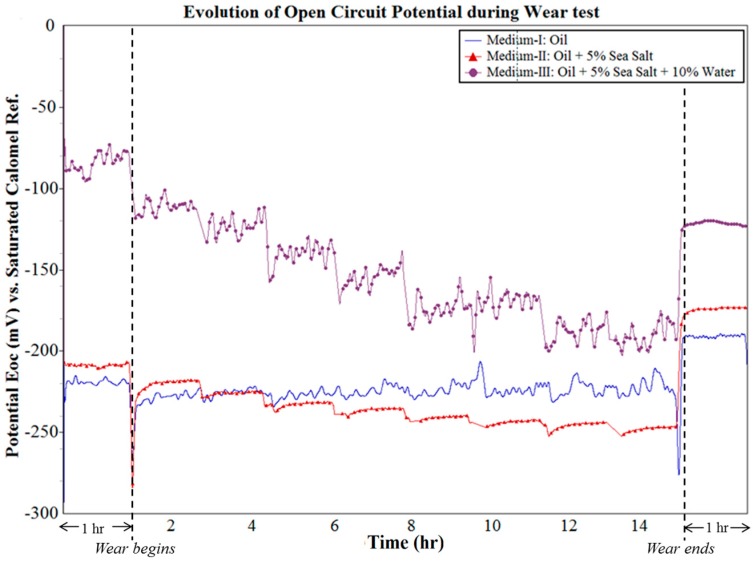
Evolution of OCP during wear test in different oil mediums.

Once the equilibrium potential was obtained at the end of 1 h of OCP measurement, the tribo-corrosion tests were initiated according to the test parameters, as detailed in Table 2 for 15 h, followed by 1 h of repassivation. The evolution of OCP before, during, and after the wear test within the system is shown in Figure 5. It can be observed that the system in contact with Medium-I was stable throughout the wear test period with minimal variation in potential. This can be attributed to the high resistance of the oil to wear and corrosion in its uncontaminated state, which is a typical characteristic of synthetic oil that was specifically formulated for application in these environments. However, in the case of wear tests performed in contaminated conditions of Medium-II and Medium-III, the OCP was observed to steadily drop from the quiescent conditions, more so in Medium-III than in Medium-II. In the case of Medium-II, the OCP drops from −210 mV to −250 mV, whereas in Medium-III, a more prominent drop from −84 mV to −200 mV was observed by the end of the wear test. In all three oil mediums, the system in contact was observed to repassivate soon after the wear test ended.

A negligible drop in potential was observed in the case of wear test in Medium-I due to negligible corrosion products expected in the medium. In the case of Medium-II, a progressive drop in the potential with time is observed. This drop clearly has distinguishable intermediate steps, which indicate that the salt contaminants in Medium-II are slowly initiating corrosion. The slow rate of corrosion initiation can be attributed to the non-polar nature of the oil. The salts remain suspended in the oil and react periodically with the steel surface. As the wear test progresses, the salts can also be expected to react with the wear debris within an oil medium in a similar manner. However, in Medium-III, there was a very high degree of drop in the potential, which indicated the formation and destruction of a large amount of corrosion products on the surface of the steel sample. The presence of water along with sea salt in Medium-III causes accelerated the corrosion of steel wear debris and wear track, which was investigated in the later stages of the analysis. The minor fluctuations in potential observed for all three curves in Figure 5 can be attributed to the cell current variations caused by the dynamic nature of the experiment, wherein the oil medium was in constant motion. This aspect of the experiment would, in fact, define the stability of the test rig design for tribo-corrosion analysis in oil medium. The influence of the dynamic nature of the experiment on the reliability of data was better understood when analyzing the potentiodynamic results in the following sections.

**Figure 6:**
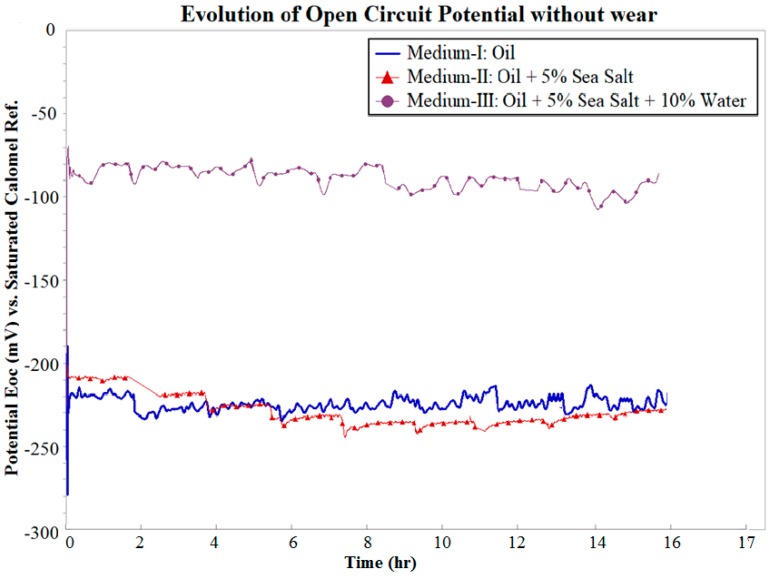
Evolution of OCP without wear.

The effects of wear on OCP during wear test were further investigated by monitoring OCP for 15 h without wear in all three mediums, as shown in Figure 6. It can be clearly observed that there was a negligible drop in OCP over this period in Mediums I, II, and III. It was estimated that over a period of 15 h, the effect of wear on the OCP was such that the OCP drops by 7 mV, 36 mV, and 98 mV in Mediums I, II, and III, respectively. These variations can be attributed to the change in the cell current flowing through the resistance of the cell solution during the wear. It can be expected that the wear debris continuously entering the cell solution during wear interact with the salt and water contaminants in the solution. This affects the stability of OCP over longer durations with progressively increasing severity.

### 3.3. Analysis of Coefficient of Friction (COF) and Wear during Tribo-Corrosion Test

**Figure 7:**
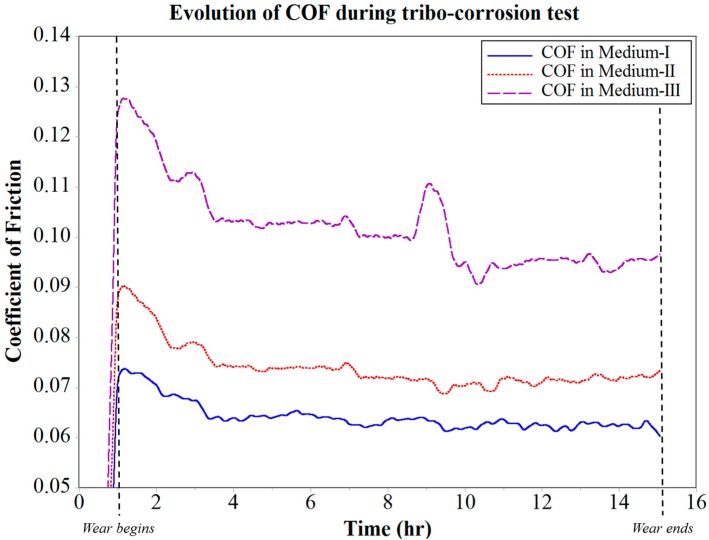
Evolution of COF during tribo-corrosion testing

The COF was monitored in situ during the tribo-corrosion test, as shown in Figure 7. It was observed that the COF of the tribo-pair in Medium-I gradually decreased over the first two hours of wear, and thereafter became stable over the remaining period of the test, with an average value of 0.066. The tribo-pair in Medium-II shows an initial drop in COF with a high slope for the first two hours as compared with that in Medium-I. This was followed by a gradual drop in COF over the following two hours, and the tribo-pair achieves an average stable COF of 0.075 after nearly 4 h of wear. The period of stability of COF in Medium-II was disrupted after 7 h of wear, and an irregular fluctuation in COF was observed thereafter till the end of the experiment. This disruption in COF can be attributed to the sea salt contaminant, which at this stage can be expected to react with the wear track, thus causing repeated formation and breakdown cycles of oxide tribo-layers.

**Figure 8:**
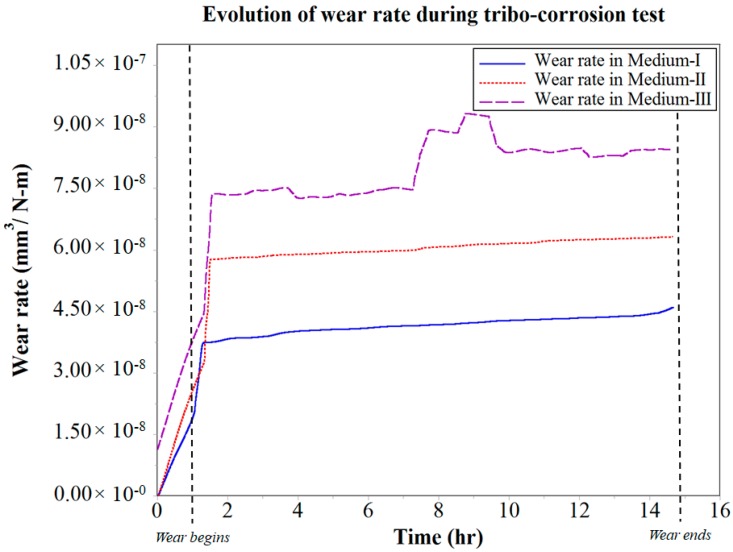
Evolution of wear rate during tribo-corrosion testing.

In the case of Medium-III, it was observed that for the first 4 h of wear, there was a high initial drop in COF with a considerably high slope when compared with Mediums I and II. This can be attributed to the wear track run-in period being constantly interrupted by the salt and water contaminants, which are immiscible in oil. At the end of the run-in period, the water contaminants can be expected to have dissolved the salt contaminants and formed an aqueous NaCl form of contaminant in the oil Medium-III. Even though the tribo-pair achieves a small duration of stable COF of 0.14 after 4 h of wear, it was observed to have been disrupted at the end of the 8 h wear period. More specifically, this disruption causes the COF to spike over the next few minutes, and then drop with similar frequency. The COF in Medium-III was consistently unstable thereafter. This behavior of COF between the tribo-pair was consistent with the observed wear rate, as seen in Figure 8. The wear rate of the tribo-pair in Medium-I and II was found to be increasing consistently with time. However, in the case of Medium-III, the disruption in COF observed after 8 h of wear was because of an increase in the wear rate. At this stage of the analysis, it was speculated that this was the point where the corrosion started to accelerate the wear occurring at the interface of the tribo-pair during sliding. This phenomenon was evidence to the tribo-corrosion in action, which was seen to affect COF and wear. Further, the effectiveness of the test rig design in analyzing and isolating the effects of tribo-corrosion in an oil medium with respect to wear and COF was also proven.

**Figure 9:**
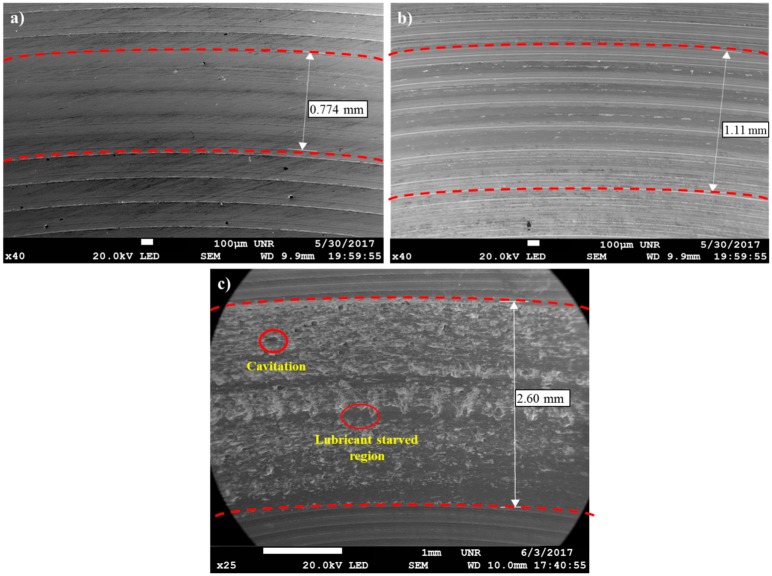
Wear track under scanning electron microscope (SEM) post tribo-corrosion testing.

The wear tracks in all three mediums were also investigated post experimentation under SEM to gauge the effect of contaminants in the oil medium on the wear track, as shown in Figure 9. It was clear that the uncontaminated condition of oil (Medium-I) was very effective and yielded a wear track that was only 0.774 mm wide, as seen in Figure 9a. Whereas the salt-contaminated oil medium (Medium-II) shows a wear track of 1.11 mm, as seen in Figure 9b. Both of these wear tracks indicated no visible anomalies along the wear track, whereas the sample in Medium-III, which was contaminated with sea salt and water, exhibited a very wide wear track, as seen in Figure 9c. It can be observed that this wear track bore evidence of cavitation due to contaminating inclusion at the tribo-pair interface, which further aided and accelerated the synergistic effects of wear–corrosion during sliding.

### 3.4. Evolution of Corrosion Rate during Tribo-Corrosion Test

**Figure 10:**
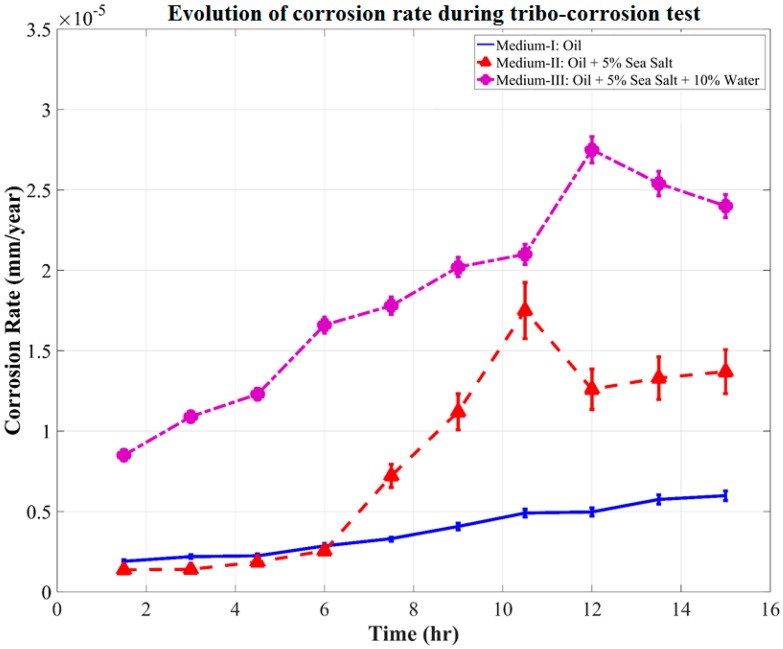
Evolution of corrosion rate during the tribo-corrosion test.

In situ direct current corrosion tests were performed in parallel with the wear test for 15 h in each of the oil mediums. During the DC corrosion testing, the potential was continuously varied from anodic condition of +0.5 V potential vs. OCP (*E_OC_*) to cathodic condition of −0.5 V potential vs. OCP (*E_OC_*) at a scan rate of 0.167 mV/s and data being sampled every second. The three-electrode DC Corrosion test rig shown in Figure 3 was based on the potentiodynamic testing module. The sequential potentiodynamic test performed follows the quantitative corrosion theory, which assumes that the rates of both anodic and cathodic processes are a function of the electron transfer reaction at the metal surface. The Butler–Volmer equation (Equation (2)) defining the opposing reactions in a corrosion system was used to determine the corrosion current (*I_corr_*), Tafel constants, and their respective Tafel curves (log *I* vs. *E* plot). The 15-h tribo-corrosion testing yielded 20 Tafel curves for each oil medium, from which the corrosion rates were calculated and plotted as a function of time, as shown in Figure 10.

(2) $$I=I_{a}+I_{c}=I_{corr}\left(e^{\frac{2.3\left(E-E_{oc}\right)}{\beta_{a}}}-e^{\frac{-2.3\left(E-E_{oc}\right)}{\beta_{c}}}\right)$$

where, *I* = measured cell current in amps; *I_a_* = current resulting from anodic reactions; *I_c_* = current resulting from cathodic reactions; *I_corr_* = the corrosion current in amps; *E* = the electrode potential; *E_OC_* = the corrosion potential in volts; *β_a_* = the anodic beta Tafel constant; *β_c_* = the cathodic beta Tafel constant.

It was observed during the tribo-corrosion test that for the first 6 h of wear, the corrosion rate in Medium-I was minimal at 0.325 × 10^−5^ mm/year. After 6 h of wear, the corrosion rate moderately accelerated over the remainder of the test and increased to a maximum of 0.689 × 10^−5^ mm/year at the end of 12 h of wear. The corrosion rate in Medium-II was similar to that in Medium-I for the first 6 h of wear, after which it linearly accelerated to a maximum of 1.792 × 10^−5^ mm/year at the end of 10.5 h of wear. This acceleration in corrosion rate in Medium-II indicates the corrosive reaction of the salt contaminants with the wear debris. Since this increase in corrosion rate did not effectively vary either the COF or the wear rate shown in Figure 7 and Figure 8, respectively, it can be attributed to the increase in the cell medium resistance that would have caused the anodic reactions to increase considerably. Hence, tribo-corrosion testing in Medium-II can be expected to yield a higher rate of wear over a prolonged wear period that would progress beyond 15 h when compared with Medium-I.

**Figure 11:**
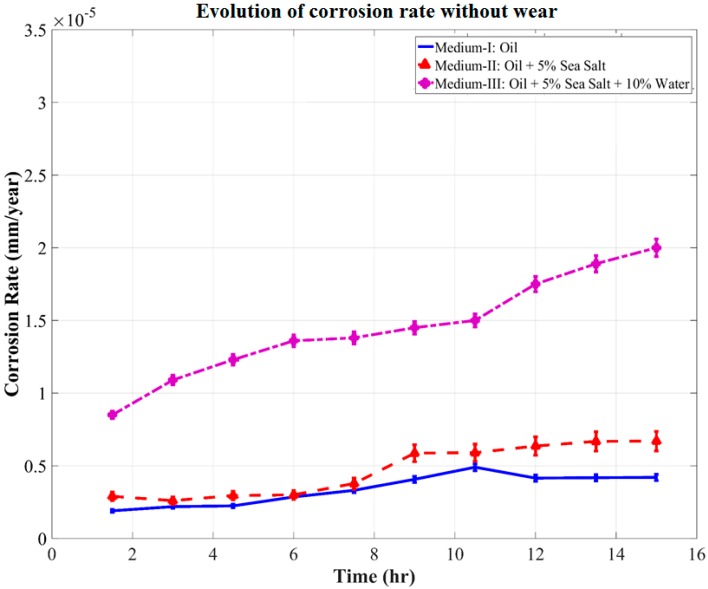
Evolution of corrosion rate without the influence of wear.

Further, in Figure 10, the corrosion rate of the system in contact with Medium-III was observed to linearly increase from the beginning of the tribo-corrosion test. The corrosion rate increases from a minimum of 0.86 × 10^−5^ mm/year at the end of 1 h of wear to a maximum of 2.58 × 10^−5^ mm/year at the end of 12 h of wear. A spike in corrosion rate was observed to be initiated after 11 h of wear, which was right after the fluctuations in COF and wear rate observed between the time periods of 8–11 h. Medium-III, which was contaminated with salt and water, seems to have stabilized as a single contaminant consisting of aqueous NaCl, which starts to corrosively react with the steel wear debris and wear track, forming corrosion elements. The corrosion elements consisting of iron oxide along with the contaminants interfere with the sliding of tribo-pair, causing cavitation, minor pitting, and starved lubrication regions at the interface, as observed in Figure 9c. It is speculated that the period during which these defects were generated led to fluctuations in the COF and wear debris, as observed between the time periods of 8–11 h. Following the generation of these corrosion inhibiting defects was the sudden spike in corrosion rate after 11 h of wear. Evidently, this was also the point at which wear and corrosion synergism effect was the maximum, because when the corrosion tests were performed without wear, the corrosion rate was fairly linear throughout for Medium-III, as can be observed in Figure 11.

In the current system, the galvanic corrosion between the contacting tribo-pair is expected to negligibly influence the corrosion rate. The phenomenon of galvanic corrosion is dependent on the ratio of cathodic and anodic surface areas, which is an important factor in the calculation of cell current density (area related cell current). This area of contact is expected to significantly influence the galvanic corrosion. Considering the tribo-pair in the present study, the electrode potentials of the two materials—alloy steel and the carbon steel disk—are identical, and minimize the extent of any possible galvanic corrosion. Still, when these electrode potentials are considered, the alloy steel will behave as the cathode, and the carbon steel will behave as the anode. Since the cathodic surface area (alloy steel: the more noble metal of the galvanic couple) is small compared with the anodic surface area (carbon steel: the less noble metal), no change in corrosion behavior will be observed. Even if the carbon steel disk is in an active state and may show the possibility of galvanic corrosion due to depassivation, the cathodic efficiency of alloy steel is much lower than that of carbon steel to effectively initiate galvanic corrosion.

**Figure 12:**
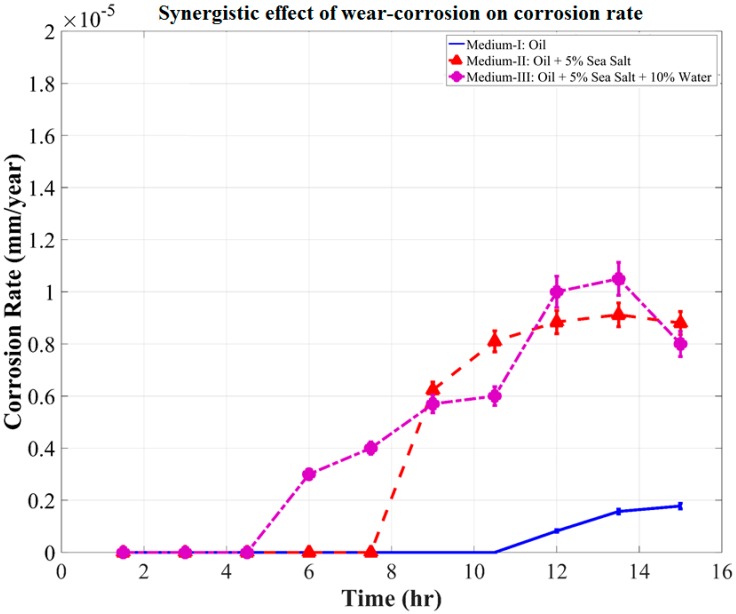
Synergistic effect of wear–corrosion on corrosion rate.

Comparing the corrosion rates shown in Figure 10 and Figure 11, the synergistic effect of wear–corrosion on corrosion rate was determined, as shown in Figure 12. This gives a clear perspective on the synergistic effect of wear–corrosion in the uncontaminated oil medium (Medium-I) and the contaminated oil mediums (Medium-II and III). The synergistic effect of wear–corrosion on corrosion rate can be observed to have been initiated after 10.5 h, 7.6 h, and 4.3 h of wear in Medium-I, II, and III, respectively. There are also observed periods of acceleration in corrosion rates in Medium-II and Medium-III, which are indicative of the breaking down of oxide tribo-layers on the surface of the sample, wear debris, and wear track. This is evidently the tribo-corrosion phenomenon that causes the variations in OCP, corrosion rate, COF, and wear rate. The repeatability test where the same set of experiments were performed twice ensured that the test rig design for analyzing this phenomenon in oil mediums was found to be effective and consistent irrespective of the contaminants and time of testing period.

## 4. Conclusions

The current study was aimed at designing and analyzing a tribo-corrosion test rig that could effectively monitor the phenomenon in an oil medium. The study attempts to solve the prolonged ineffectiveness of tribo-corrosion measurements in an oil medium due to the design and cell medium limitations. It was found that the kinetic nature of the oil medium during testing causes moderate fluctuation in the reading, which can be compensated by strategically aligning the reference and counter electrodes with the wear track and fluid flow. It was observed that the synergistic effect of wear and corrosion in an uncontaminated oil medium was negligible. Whereas, in the oil mediums that were contaminated with salt and water, the effect of tribo-corrosion was very prominent. When the oil medium was contaminated with 5 wt.% salt, the effect of tribo-corrosion on corrosion rate was observed after 7 h, while in the oil contaminated with 5 wt.% salt and 10 wt.% water, it was observed as early as 3 h into the tribo-corrosion test. The test rig design proves to be an effective method of monitoring wear and corrosion synergism in an oil medium, which can be in contaminated or uncontaminated condition. In addition, this study will enable future research in terms of investigating oil life in correlation to the synergistic effects of wear and corrosion rates while the electrolyte is continuously agitated.

The in situ oil monitoring system presented in the current study has a wide range of industrial applicability involving oil and lubricating systems, such as transmission systems and engines in automobiles, pressure vessel cooling systems, wind turbines, heavy duty drills, and a variety of industrial machinery. Further, the system will enable better control and monitoring of over and under usage of oils and lubricants, which will reduce the overall oil waste output into our ecosystem.

## Figures and Tables

**Table 1:** Specifications of commercial grade automatic transmission fluid (ATF) used as the lubricating medium.

Oil Specifications	Values
Mass density at 15 °C	867 kg/m^3^
Colour, ASTM	6.5
Viscosity at 40 °C	32.26 mm^2^/s
Viscosity at 100 °C	7.1 mm^2^/s
Viscosity index	190
Viscosity at 40 °C	12,600 cP
Flash point	183 °C
Pour Point	−48 °C

**Table 2:** Test parameters of the tribo-corrosion measurement.

Test Parameters	Value
Normal Force	10 N
Rotation speed	50 rpm
Duration of wear	15 h
Wear track radius	15 mm
Ball diameter	6.5 mm
Electrolyte/Lubricant medium	Commercial grade (refer Table 1)
Temperature	24 °C (room)
Humidity ratio	10% RH

**Table 3:** Experimental designs aimed at analyzing the effectiveness of the retrofitted tribo-corrosion test rig and investigating the tribo-corrosion in Dextron III oil medium.

Stage	Objective	Experiments	Data Recorded
Stage-I	Establish control wear rate.	Ball-on-disk test in oil medium without corrosion cell.	Wear rate in oil medium.
Stage-II	Establish control for the synergistic effect of wear and corrosion without any external contaminants.	Ball-on-disk test in oil medium with corrosion cell. Static corrosion cell experiment in oil without wear, to isolate corrosion rate.	Synergistic effect of wear and corrosion in oil medium.
Stage-III	Effect of one external contaminant in the oil medium on the synergism of wear and corrosion.	Ball-on-disk test in oil +5% (wt.%) sea salt-medium with corrosion cell. Static corrosion cell experiment in oil +5% (wt.%) sea salt medium without wear, to isolate corrosion rate.	Synergistic effect of wear and corrosion in oil medium consisting of oil +5% (wt.%) sea salt.
Stage-IV	Effect of two external contaminants in the oil medium on the synergism of wear and corrosion.	Ball-on-disk test in oil +5% (wt.%) sea salt +10% (wt.%) water medium with corrosion cell. Static corrosion cell experiment in oil +5% (wt.%) sea salt +10% (wt.%) water medium without wear, to isolate corrosion rate.	Synergistic effect of wear and corrosion in oil medium +5% (wt.%) sea salt +10% (wt.%) water.

**Table 4:** Oil mediums and the contaminants used for tribo-corrosion experiments.

Oil Medium	Contaminate Introduce	Oil Contents
Medium-I	-	oil (uncontaminated)
Medium-II	5% (wt.%) sea salt	oil contaminated with sea salt
Medium-III	5% (wt.%) sea salt and 10% (wt.%) water	oil contaminated with sea salt and water
